# 1579. Incidence and Predicting Factors of Opportunistic Infections after Initiation of Antiretroviral Therapy: A Retrospective Cohort Study

**DOI:** 10.1093/ofid/ofad500.1414

**Published:** 2023-11-27

**Authors:** Prapon Wongkittipong, Sasisopin Kiertiburanakul

**Affiliations:** Faculty of Medicine Ramathibodi Hospital, Mahidol University, Bangkok, Krung Thep, Thailand; Faculty of Medicine Ramathibodi Hospital, Mahidol University, Bangkok, Krung Thep, Thailand

## Abstract

**Background:**

Antiretroviral therapy (ART)-associated opportunistic infections (OIs) is the term proposed to encompass all OIs diagnosed after ART initiation, and it is not uncommon. We aimed to determine the incidence of OIs and the predicting factors for OIs developing after ART initiation among Thai people living with HIV (PLHIV).

**Methods:**

In the university hospital setting, a retrospective cohort study was conducted among naïve PLHIV who initiated ART between January 2016 and December 2019.

**Results:**

A total of 401 PLHIV were included in the analysis. Of these, the mean (SD) age was 37.1 (11.9) years, 74.3% were male, and 57.6% had heterosexual risk. There were 247 (61.6%) diagnosed with AIDS and a median (IQR) CD4 count at ART initiation was 166 (49-314) cells/µL. The most common prior OIs were tuberculosis (26.4%) and *Pneumocystis jirovecii* pneumonia (11.5%). Of all, 38 (9.5%) PLHIV developed OIs after initiating ART with an incidence rate of 25.6/1000 person-years. The median time (IQR) from ART initiation to the OIs occurrence was 26.5 (14-73) days. PLHIV who develop OI after ART initiation were more likely to have lower mean body mass index (BMI) (20.1 vs 22.3 kg/m^2^), lower median CD4 counts (36 vs 188 cells/µL), lower mean hemoglobin levels (10.8 vs 12.4 mg/dL), a higher proportion of AIDS diagnosis (86.5% vs 57.9%), having prior OIs (78.9% vs 35.5%), and a higher proportion of initiation with dolutegravir (DTG)-based regimen (10.5% vs 3%) (p < 0.05, all). By multivariate Cox proportional hazard regression, having BMI ≤18.5 kg/m^2^ (aHR 2.28; 95% CI 1.18-4.42, p=0.015), symptomatic at presentation (aHR 13.59; 95% CI 3.24-56.9, p < 0.001), SGPT >55 U/L (aHR 2.09; 95% CI 1.06-4.15, p=0.035), and initiation with DTG-based regimen (aHR 4.39, 95% CI 1.54-12.48, p=0.006) were statistically significant associated OIs after ART initiation. The OI-associated mortality was 5.3%.

**Conclusion:**

The OIs after ART initiation are not uncommon. Malnutrition, symptomatic at presentation, abnormal liver enzyme, and initiation with DTG-based regimens is predicting factors of OI occurrences. It is crucial for physicians to monitor and appropriate treatment of OIs after ART initiation.

**Disclosures:**

**All Authors**: No reported disclosures

